# Computational methods reveal novel functionalities of PIWI-interacting RNAs in human papillomavirus-induced head and neck squamous cell carcinoma

**DOI:** 10.18632/oncotarget.23464

**Published:** 2017-12-19

**Authors:** Aswini R. Krishnan, Yuanhao Qu, Pin Xue Li, Angela E. Zou, Joseph A. Califano, Jessica Wang-Rodriguez, Weg M. Ongkeko

**Affiliations:** ^1^ Department of Otolaryngology-Head and Neck Surgery, University of California San Diego, La Jolla, California, USA; ^2^ Veterans Administration San Diego Healthcare System and Department of Pathology, University of California San Diego, La Jolla, California, USA

**Keywords:** piRNA, HNSCC, ncRNA, HPV

## Abstract

Human papillomavirus (HPV) infection is the fastest growing cause of head and neck squamous cell carcinoma (HNSCC) today, but its role in malignant transformation remains unclear. This study aimed to conduct a comprehensive investigation of PIWI-interacting RNA (piRNA) alterations and functionalities in HPV-induced HNSCC. Using 77 RNA-sequencing datasets from TCGA, we examined differential expression of piRNAs between HPV16(+) HNSCC and HPV(–) Normal samples, identifying a panel of 30 HPV-dysregulated piRNAs. We then computationally investigated the potential mechanistic significances of these transcripts in HPV-induced HNSCC, identifying our panel of piRNAs to associate with the protein PIWIL4 as well as the RTL family of retrotransposon-like genes, possibly through direct binding interactions. We also recognized several HPV-dysregulated transcripts for their correlations with well-documented mutations and copy number variations in HNSCC as well as HNSCC clinical variables, demonstrating the potential ability of our piRNAs to play important roles in large-scale modulation of HNSCC in addition to their direct, smaller-scale interactions in this malignancy. The differential expression of key piRNAs, including *NONHSAT077364*, *NONHSAT102574*, and *NONHSAT128479*, was verified *in vitro* by evaluating endogenous expression in HPV(+) cancer vs. HPV(–) normal cell lines. Overall, our novel study provides a rigorous investigation of piRNA dysregulation in HPV-related HNSCC, and lends critical insight into the idea that these small regulatory transcripts may play crucial and previously unidentified roles in tumor pathogenesis and progression.

## INTRODUCTION

Although tobacco use and alcohol consumption are the well-established and major risk factors for head and neck squamous cell carcinoma (HNSCC), high-risk human papilloma virus (HPV) infection, primarily due to HPV16, is quickly emerging as the fastest growing cause of the disease [1]. Over the past few decades, the proportion of HPV-associated HNSCCs has steadily risen to comprise 10–30% of all cases of HNSCC, while specific subtypes including oropharyngeal squamous cell carcinoma (OPSCC) display a corresponding proportion of around 40–80% [1]. Recent studies have suggested HPV infection to increase risk of HNSCC up to 22-fold [2].

Despite the prevalence of HPV-associated HNSCC, current knowledge of its molecular mechanism of pathogenesis and progression is limited. After demonstration of the carcinogenic role of HPV in HNSCC in 2000 [3], studies have implicated HPV16 *E6/E7* gene expression in promoting malignant transformation in HNSCC [4]. The over-expressed E6 onco-protein caused by HPV16 is able to induce degradation of the tumor suppressor protein p53 via the ubiquitin pathway [4]. At the same time, E7 onco-protein can bind and inactivate the retinoblastoma (Rb) tumor suppressor gene product, promoting cell G1-S phase transition [4]. Aside from the effects of E6/E7 on these tumor suppressors, HPV-induced alterations in other factors, including genomic stability and epigenetics, are also known to play critical roles in malignant transformation and tumor progression.

Advances in the understanding of non-coding RNAs (ncRNAs) have revealed their active involvement in a myriad of biological processes including transcriptional and post-transcriptional regulation. Non-coding RNAs have also been implicated in a number of cancers [5–7]. As the largest class of small non-coding RNAs, PIWI-interacting RNAs (piRNAs) are thought to function in cells by forming piRNA/PIWI protein complexes that promote retrotransposon silencing and post-transcriptional regulation [8, 9]. Interestingly, our previous study on the non-coding landscape of HNSCC indicated that piRNAs dysregulated in HNSCC may have associations with different etiological factors [10]. Our follow-up study on smoking-induced piRNA alterations identified a novel panel of piRNAs specifically associated with smoking-induced HNSCC [11]. Therefore, in this study, we attempted to comprehensively analyze the HPV-associated piRNA landscape of HNSCC.

Using 77 HPV16(+) and HPV(–) RNA-seq datasets from the Cancer Genome Atlas (TCGA), we identified HPV-specific piRNA dysregulation in HNSCC. We then analyzed the mechanistic and clinical relevancies of these piRNAs in HPV-induced head and neck malignancies to evaluate their overall functional significances in tumor pathogenesis and progression.

## RESULTS

### Identification of HPV-dysregulated piRNAs in HNSCC

**Figure 1:**
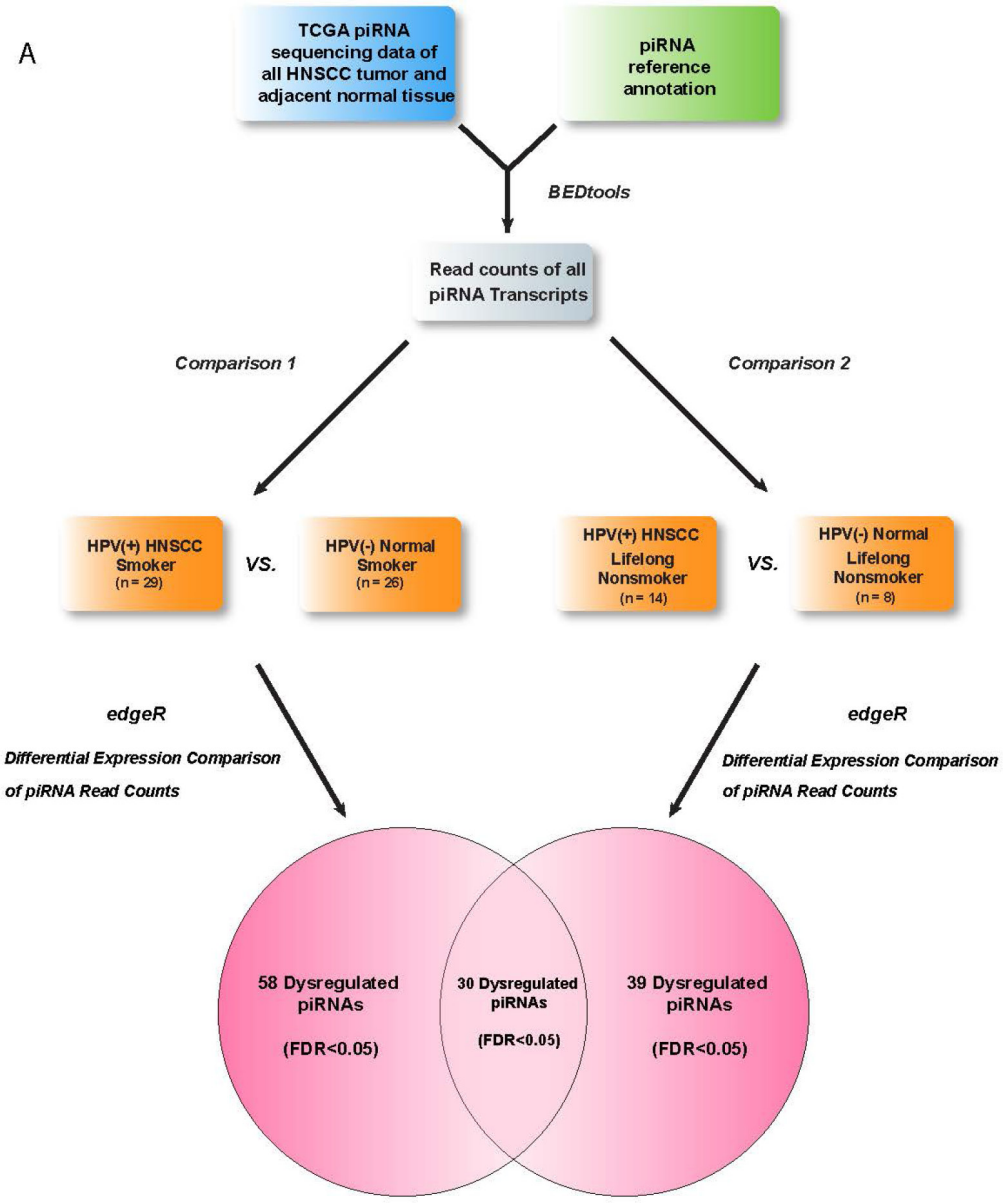
Schematic detailing the RNA-seq analysis pipeline used to identify HPV-associated piRNA candidates (*p* < 0.05, FDR < 0.05)

**Figure 2:**
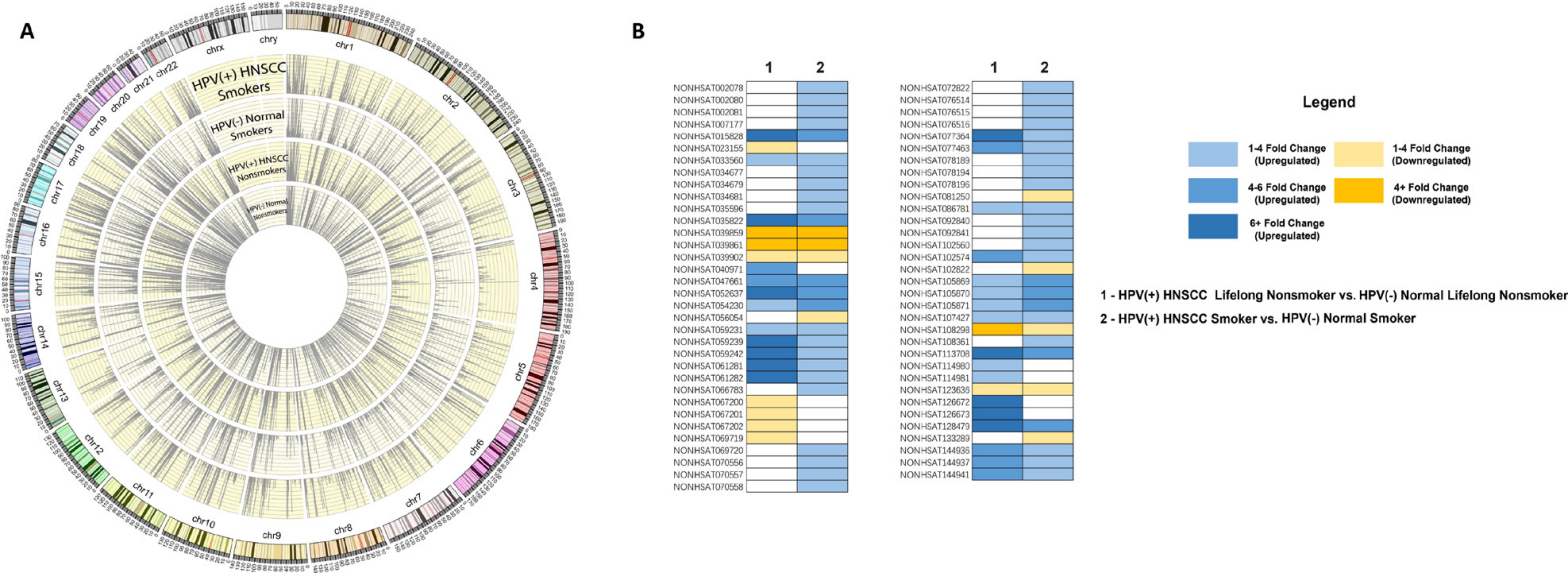
(**A**) Circos plot depicting piRNA expression in four cohorts: HPV(+) Cancer Smokers, HPV(–) Normal Smokers, HPV(+) Cancer Nonsmokers, and HPV(–) Normal Nonsmokers. (**B**) Similarities and differences in piRNA dysregulation in two comparisons: HPV(+) HNSCC Nonsmokers vs. HPV(–) Normal Nonsmokers and HPV(+) HNSCC Smokers vs. HPV(–) Normal Smokers. piRNA dysregulation is color-coded by fold change. Boxes with no color indicate that the piRNA was not found dysregulated in that comparison.

In order to identify HPV-associated piRNAs in HNSCCs, we examined 77 HPV16(+) HNSCC and HPV(–) Normal RNA-seq datasets from TCGA with available clinical data (dataset IDs in Supplementary Table 1). We used negative-binomial based differential expression testing to perform 2 comparisons of piRNA expression between HPV16(+) and HPV(–) cohorts, separately analyzing smoking and nonsmoking datasets as smoking has been suggested to independently alter piRNA expression in HNSCC (Figure 1) [11]. We identified 58 piRNAs to be dysregulated between HPV16(+) HNSCC Smokers and HPV(–) Normal Smokers and 39 piRNAs to be dysregulated between HPV16(+) HNSCC Nonsmokers and HPV(–) Normal Nonsmokers (FDR < 0.05, Supplementary Table 2). The differential dysregulation of piRNAs due to HPV in smoking and nonsmoking backgrounds is displayed in Figure 2.

In order to perform a robust identification of HPV-altered piRNAs, we selected only piRNAs dysregulated in both comparisons to include in our panel of HPV-dysregulated transcripts, identifying 30 such candidates (Figure 1, Supplementary Table 2).

### Associations of HPV-dysregulated piRNAs with PIWI proteins

**Figure 3:**
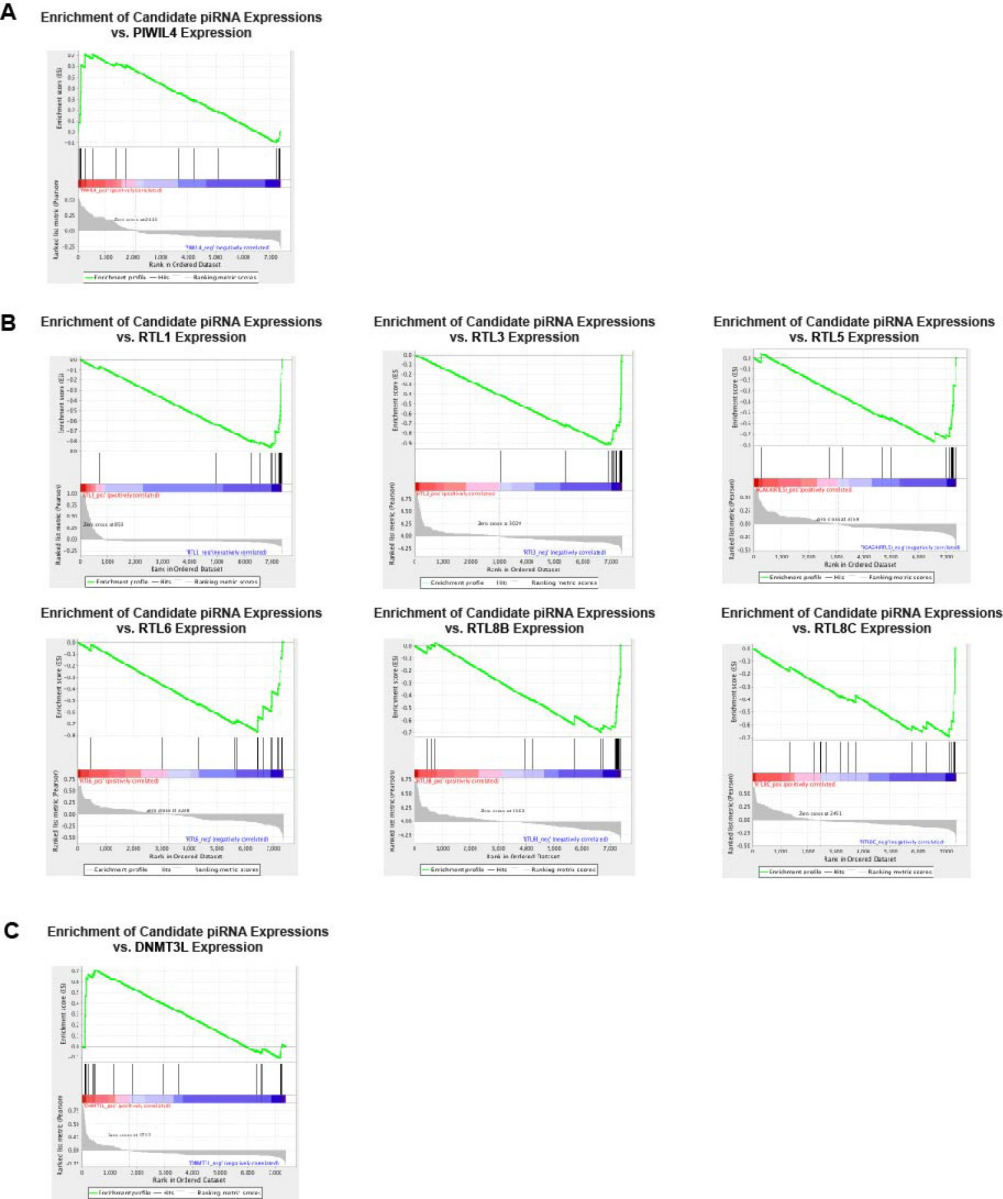
HPV-dysregulated piRNAs display (**A**) significant positive enrichment relative to PIWIL4 expression, (**B**) significant negative enrichment relative to RTL gene expression, and (**C**) significant positive enrichment relative to DNMT3L expression.

piRNAs are thought to execute their functionality by associating with PIWI proteins, forming PIWI-piRNA complexes that promote gene silencing [12]. Therefore, utilizing gene set enrichment analysis (GSEA), we attempted to identify PIWI protein candidates that may potentially associate with piRNAs in HPV-associated HNSCCs. Ranking 7385 piRNAs containing expression counts in at least one patient in order of positive to negative correlation with PIWI protein expression, we evaluated all piRNAs in our set of 30 HPV-dysregulated piRNAs for significant enrichment, separating upregulated (*n* = 24) and downregulated (*n* = 6) transcripts in order to match enrichment results to direction of dysregulation in cancer. We found our HPV-upregulated piRNA set to be positively enriched relative to *PIWIL4* expression in HNSCC, suggesting a positive correlation between upregulated piRNAs expression and *PIWIL4* expression (Figure 3A).

### Potential regulation of the RTL family by HPV-dysregulated piRNAs

Recent studies suggest that PIWI-piRNA complexes promote retrotransposon silencing either by targeting mRNA transcripts or mediating DNA methylation [12]. In order to more thoroughly understand the role of our HPV-dysregulated piRNAs in retrotransposon silencing, we decided to conduct a two-step analysis.

First, we assessed relationships between HPV-related piRNA expression and retrotransposon and retrotransposon-like gene expression to identify potential targets of our piRNAs. Utilizing GSEA, we identified our set of HPV-upregulated piRNAs to be significantly negatively enriched relative to the expression of various members of the retrotransposon-derived family RTL (FDR < 25%, Figure 3B). This data suggests an inverse relationship between HPV-dysregulated piRNA expression and RTL family expression, an expected result for piRNA-induced repression. Moreover, we identified our piRNAs to be positively enriched with respect to the expression of DNMT3L, a protein essential for both proper transposon methylation and repression [13], suggesting that piRNA-induced repression may be potentially mediated by a methylation-related mechanism, as mentioned above (Figure 3C).

**Figure 4:**
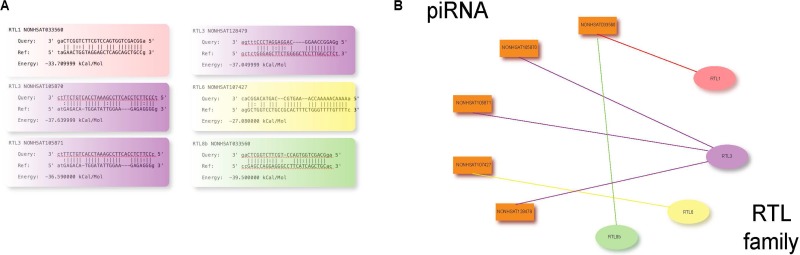
(**A**) miRanda target prediction reveals the potential for direct binding interactions between specific piRNAs and RTL family genes. (**B**) Conceptual map of miRanda-predicted piRNA-RTL binding interactions.

Next, to verify the potential of our piRNAs to directly silence these genes, we explored the presence of direct binding interactions between specific piRNAs and RTL family members. Employing the miRanda algorithm, we determined all GSEA-identified RTL members except RTL5 and RTL8A to be predicted binding targets of specific PIWI-piRNA complexes (Figure 4A), with these interactions mapped in Figure 4B.

### Associations of piRNAs with HNSCC-associated genomic alterations

**Figure 5:**
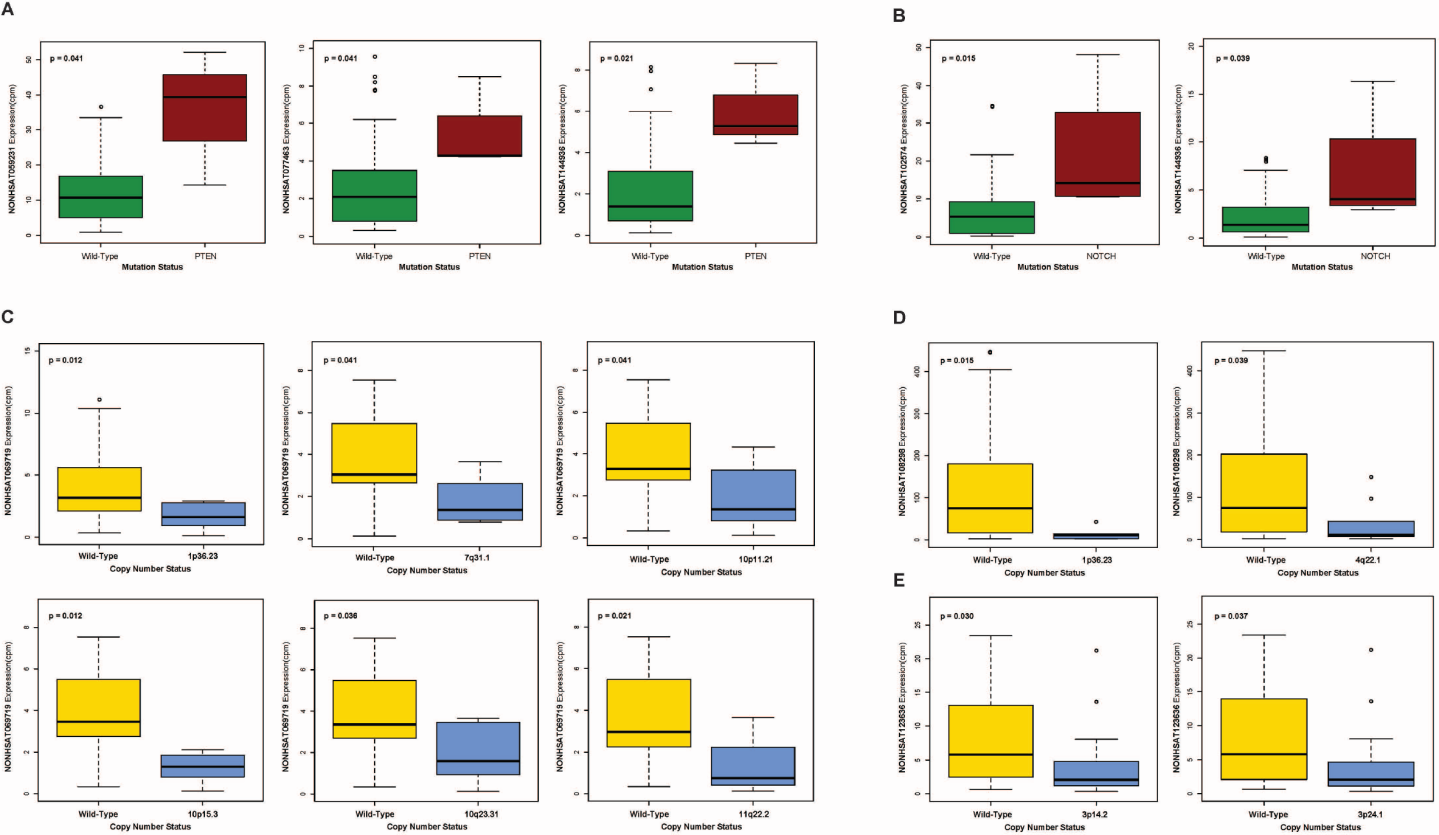
(**A**–**B**) Boxplots correlating expression of select HPV-dysregulated piRNAs to (A) PTEN mutation and (B) NOTCH mutation. (**C**–**D**) Boxplots correlating expression of select HPV-dysregulated piRNAs, (C) NONHSAT069719, (D) NONHSAT108298, and (**E**) NONHSAT123636, to common copy number variations in HNSCC (Wilcoxon rank sum, *p* < 0.05).

In order to explore the larger mechanistic roles of our piRNAs in HNSCC, we correlated expression of all 30 HPV-dysregulated piRNAs to frequent somatic mutations and copy number variations (CNV) documented in HNSCC. Employing the Wilcoxon rank-sum test (*p* < 0.05), we identified upregulated piRNAs *NONHSAT059231*, *NONHSAT077463*, and *NONHSAT144936* to display elevated expression in the presence of *PTEN* mutation (Figure 5A) and *NONHSAT102574* and *NONHSAT144936* to exhibit increased expression in the presence of *NOTCH* mutation (Figure 5B). Finally, we identified downregulated piRNAs *NONHSAT069719*, *NONHSAT108298*, and *NONHSAT123636* to exhibit significantly lowered expression in the presence of a variety of HNSCC-associated CNVs (Figure 5C–5E).

### Clinical significances of HPV-dysregulated piRNAs

**Figure 6:**
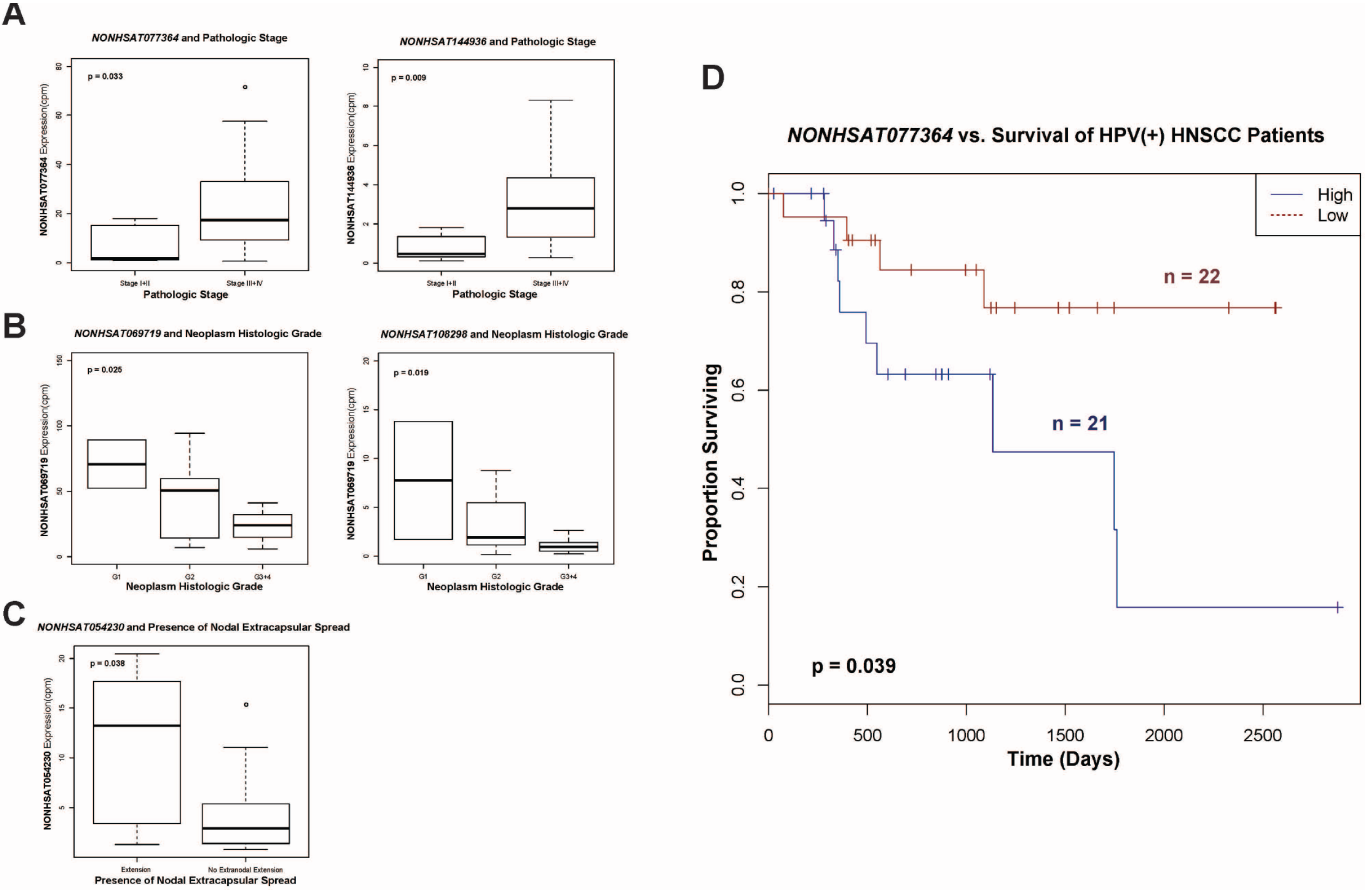
Boxplots indicating (**A**) upregulation of piRNAs NONHSAT077364 and NONHSAT144936 with higher pathologic stage, (**B**) dowregulation of piRNAs NONHSAT069719 and NONHSAT108298 with higher histologic grade, and (**C**) upregulation of NONHSAT054230 with presence of nodal extracapsular spread. (**D**) Kaplan-Meier curve depicting survival outcomes based on relative high and low expression of NONHSAT077364.

Finally, we examined the clinical relevancies of all 30 HPV-dysregulated piRNAs, using the Kruskal-Wallis test (*p* < 0.05) to assess their expression relative to a range of HNSCC clinical features. Among all HPV16(+) patients with available clinical data (*n* = 43), we found upregulated piRNAs *NONHSAT077364* and *NONHSAT144936* to display significantly elevated expression with higher pathologic stage (Figure 6A). Downregulated transcripts *NONHSAT069719* and *NONHSAT108298* displayed significantly decreased expression with higher histologic grade (Figure 6B). Upregulated piRNA *NONHSAT054230* displayed significantly increased expression in presence of nodal extracapsular spread (Figure 6C). We identified *NONHSAT077364* to be significantly predictive of patient outcome in both univariate and multivariate Cox regression analyses (Figure 6D, Supplementary Table 4).

### *In vitro* validation of HPV-induced piRNA dysregulation

**Figure 7:**
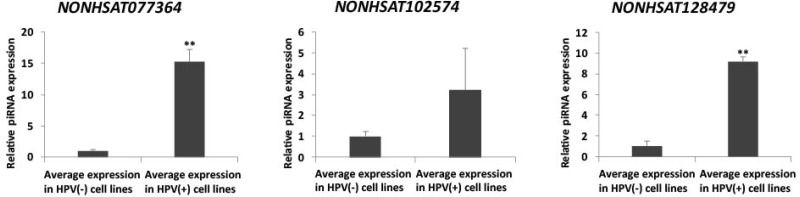
qRT-PCR verifies dysregulation of NONHSAT077364, NONHSAT102574, and NONHSAT128479 in HPV(+) cancer vs. HPV(–) normal cell lines. (Student’s *t*-test; ^**^p < 0.01)

To verify the clinical dysregulation of our HPV-associated panel of piRNAs, we examined their endogenous expression in HPV(+) squamous cell carcinoma cell lines UM-SCC-47 and 93-VU-147T in comparison to their expression in HPV(–) normal human epithelial cell lines HaCaT and OKF6. We identified the piRNAs *NONHSAT077364*, *NONHSAT102574*, and *NONHSAT128479*, recognized for their clinical and mechanistic significances in HPV(+) HNSCC, to display their expected dysregulation in HPV(+) cancer vs. HPV(–) normal cell lines, in support of our computational findings (Figure 7).

## DISCUSSION

HPV infection is the fastest growing cause of HNSCC today [1]. In this study, we conducted a comprehensive investigation of the clinical and mechanistic roles of piRNAs in HPV-associated HNSCCs.

In order to perform a robust identification of HPV-associated piRNAs, we analyzed piRNA differential expression between 77 HPV16(+) HNSCC and HPV(–) normal RNA-seq datasets from TCGA, separately evaluating HPV-induced piRNA dysregulation in smokers and in nonsmokers. We filtered for only the commonly dysregulated transcripts to eliminate the confounding effects of smoking status, and identified 30 HPV-altered piRNAs common between HPV(+) HNSCC and HPV(–) normal comparisons of smokers and nonsmokers.

Recent studies suggest that piRNAs execute their functionality by associating with PIWI proteins (PIWIL1-PIWIL4), forming PIWI-piRNA complexes that play various regulatory roles [9, 12, 14]. Investigating relations between HPV-altered piRNAs and PIWI proteins, we identified our set of HPV-upregulated piRNAs in HNSCC to be positively correlated with PIWIL4 expression, suggesting that they may potentially interact with PIWIL4. While PIWIL4 remains relatively unexplored in cancer, past findings suggest that it is upregulated in breast cancer and that it promotes cell migration, decreases apoptosis, and mildly increases proliferation, although whether or not these events occur through a piRNA-mediated mechanism remains unclear [15].

PIWI-piRNA complexes are thought to be primarily involved in targeted gene silencing, particularly retrotransposon silencing, through two primary mechanisms. First, the complexes may promote DNA methylation and therefore silencing of transposable elements by binding to transposon sequences (through piRNA-DNA complementarity) and guiding DNA methyltransferases to these sequences [16, 17]. Our analyses indicated a positive correlation in expression between HPV-upregulated piRNAs and DNMT3L, a protein essential for DNA methylation [13], suggesting that this mechanism may be important in piRNA-mediated retrotransposon silencing. Second, the PIWI-piRNA complex may post-transcriptionally silence genes, including, but not limited to, retrotransposons, by direct binding of the piRNA to specific mRNA sequences, followed by PIWI protein-mediated cleavage or deadenylation and degradation of the mRNA [18].

As the specific interactions between piRNAs and retrotransposons in any malignancy remain virtually unexplored, we investigated the relationship between HPV-altered piRNAs and retrotransposon and retrotransposon-like gene expression in HNSCC. We determined a negative correlation between HPV-upregulated piRNA expression and expression of six genes in the retrotransposon-like family RTL, matching our expectations from piRNA-induced repression. While the RTL family itself is not comprised of retrotransposons, previous studies suggest RTL genes to share homology with Ty3/gypsy retrotransposons but to have lost their ability to retrotranspose, suggesting that RTL genes may have structural similarities to retrotransposons that allow them to be targeted by piRNAs [19, 20]. piRNA target analysis suggested specific HPV-upregulated piRNAs to display significant sequence complementarily to all except two of the six correlation-relevant RTL genes, namely RTL1, RTL3, RTL6, and RTL8B. Interestingly, RTL genes seem to play opposing roles in carcinogenesis. RTL6 is a close derivative of RTL7 which has been recognized for its downregulation in pancreatic and gastric cancers and therefore likely acts as a tumor suppressor [21]. By contrast, RTL1 has been documented for its upregulation in hepatocellular carcinoma which suggests that it functions as an oncogene [22]. This suggests that while piRNAs may suppress RTL gene expression through either of the two mechanisms described above, more potent regulators may interfere with or oppose these interactions.

Next, we attempted to explore the role of our piRNAs in the bigger context of HPV-related HNSCC pathogenesis, examining correlations of HPV-dysregulated piRNAs with common mutations and copy-number variations in HNSCC. We identified *NONHSAT059231, NONHSAT077364*, and *NONHSAT144936* to associate with mutations in the tumor suppressor gene *PTEN*, a negative regulator of the Akt pathway that suppresses apoptosis and increases cell survival and has been found downregulated in 30% of HNSCCs [23]. Reduced expression of HPV-downregulated piRNA *NONHSAT069719* also correlated with deletion of *10q23.3*, the locus of the *PTEN* gene [24]. Interestingly, PTEN deficiency has been associated with an HPV-mediated mechanism, whereby HPV16 activates EGFR resulting in PTEN inactivation, which may in part be modulated by our piRNAs [25, 26].

In addition, *NONHSAT102574* and *NONHSAT 144936* associated with mutations in *NOTCH*, a gene known to function primarily as a tumor suppressor in HNSCC [27]. In particular, loss-of-function mutations in *NOTCH* have been found to generate truncated proteins lacking the ankyrin repeat domain, crucial for transactivation for target genes and thought to contribute to HNSCC progression [28]. *NONHSAT069719* and *NONHSAT123636* both correlated with amplification of *1q36.2*, the locus of *mTOR*, an oncogene that has frequently been found upregulated in HNSCCs [29, 30]. Overactivation of the Akt pathway, perhaps due to loss of the regulatory functions of PTEN as mentioned above, may also play a role in mTOR overactivation, as mTOR is a downstream effector of Akt, and may further contribute to tumorigenesis. While the exact mechanisms by which piRNAs associate with well-documented HNSCC-associated aberrations remains to be elucidated, our findings suggest piRNAs to have far-reaching roles in HPV-induced tumorigenesis going well-beyond the implications of their direct, smaller-scale interactions.

We examined the clinical significances of our HPV-altered piRNAs in HNSCC to evaluate whether they have large-scale relevancies to tumor pathogenesis and progression. We found *NONHSAT077364* and *NONHSAT144936* to associate with pathologic stage, *NONHSAT069719* and *NONHSAT108298* with histologic grade, and *NONHSAT054230* with nodal extracapsular spread, in a manner consistent with their direction of dysregulation in HPV-associated HNSCCs. These findings suggest that directionally specific piRNA alterations are significant in tumor pathogenesis and progression, either by playing a role in cancer induction or being a result of cancer induction. Our previous study of smoking-induced piRNA dysregulation in HNSCC implicated *NONHSAT077364*, *NONHSAT069719*, and *NONHSAT108298* in smoking-induced oncogenesis as well, suggesting that these piRNAs may be generally implicated in HNSCCs [11]. To the best of our knowledge, all 5 transcripts remain previously unrecognized for clinical functionality in any malignancy, and *NONHSAT144936* and *NONHSAT054230* are completely novel. Lastly, we found *NONHSAT077364*, *NONHSAT102574*, and *NONHSAT128479* to be consistently upregulated in HPV(+) cancer vs. HPV(–) normal cell lines, consistent with our expected clinical dysregulation, verifying the potential of these transcripts to significantly modulate HPV-related HNSCC.

At present, rigorous studies on PIWI-interacting RNA function are lacking, with significantly limited knowledge on their functionality in HPV-induced HNSCC or mechanistic involvement in malignant transformation. We believe our findings to shed novel insights into crucial roles of piRNAs in regulating both small- and large-scale cellular interactions and ultimately modulating clinical phenotypes in HPV-induced HNSCC.

## MATERIALS AND METHODS

### RNA-seq datasets and clinical data

MapSplice-aligned TCGA BAM files were obtained from the UCSC Cancer Genomics Hub (https://cghub.ucsc.edu/) on 11 March 2015. To investigate piRNA expression, we downloaded RNA-seq datasets for all 77 HPV16(+) HNSCC and HPV(–) Normal head and neck tissue samples. The TCGA barcodes for all patients whose datasets were used in this study are provided in Supplementary Table 1.

Patient clinical data, including smoking status, were downloaded from the TCGA Data Portal on 20 March 2015 (https://tcga-data.nci.nih.gov/tcga/findArchives.htm).

### piRNA expression analysis

piRNA read counts were generated from sequencing datasets via BEDtools coverageBed (https://github.com/arq5x/bedtools2) using piRNA annotation files. The piRNA BED file containing 27,127 piRNA transcripts was obtained from NONCODEv4 (http://www.bioinfo.org/NONCODEv4/), a database integrating ncRNA data from RefSeq, Ensembl, and published literature. Read count tables were imported into edgeR v3.0 (http://www.bioconductor.org/packages/release/bioc/html/edgeR.html), and lowly expressed piRNAs were filtered from the analysis. Following TMM normalization, pairwise comparisons were applied to identify significantly differentially expressed piRNAs between HPV16(+) and HPV(–) patient cohorts, as visualized in Figure 1A.

### Association of ncRNA expression with tumor mutations and copy number aberrations

TCGA tumor mutation calls were obtained from mutation annotation files (maf) generated by the Broad Institute GDAC Firehose on 20 August 2016. We focused our analysis on 26 most frequently mutated genes in HNSCCs, as determined by whole exome sequencing of an independent tumor cohort by Stransky *et al*. (Supplementary Table 3A) [28]. Wilcoxon rank sum tests were employed to test for significant associations between piRNAs expression level (cpm) and mutational status.

Copy number variations for the TCGA tumors were obtained from the GISTIC2 pipeline in Firehose on 20 August 2016. Similarly, 73 significant (99% confidence) focal amplifications and deletions, along with all amplifications on *3q26*, *8q24*, and *11q13* (the most frequent CNVs in HNSCC), were analyzed for correlation to piRNAs using Wilcoxon rank sum tests (Supplementary Table 3B) [31].

### Gene Set Enrichment Analysis (GSEA) to identify piRNA associations with mRNA expression

Available mRNA data for all HPV(+) HNSCC patients (*n* = 41) was obtained from TCGA on 2016 June 28. The GSEA software from the Broad Institute (http://www.broad.mit.edu/gsea/) was used to identify enrichment of piRNA expression in relation to various mRNAs in all HPV(+) patients. The continuous mRNA expression values were used as phenotype labels, while the full set of 7385 piRNAs with available expression data were used to form the ranked list. Upregulated (*n* = 24) and downregulated (*n* = 6) HPV-altered piRNAs were modeled as separate gene sets, independently examined for enrichment with respect to each mRNA. FDR < 25% is considered statistically significant as per GSEA [32].

### miRanda identification of piRNA targets

piRNA targets were identified by first determining potential target sites using sequence complementarity between each piRNA and the mRNA of interest, followed by an estimation of thermodynamic stability of RNA duplexes based on these alignments, by the miRanda algorithm (v 3.3a) (http://www.microrna.org/microrna), applying stringent alignment score (sc; ≥ 170) and energy threshold (en; ≤ –20.0 kcal/mol) [33].

### Association of piRNA expression with clinical covariates and patient survival

Employing the Kruskal-Wallis test, we correlated piRNA expression to clinical variables using clinical data and piRNA expression values (in cpm), independently analyzing piRNA correlations in HPV16(+) HNSCC patients, HPV16(+) Smoking patients, and HPV16(+) Nonsmoking patients. Patients with no available information for a given characteristic were filtered from analyses involving that variable.

Candidate piRNAs were associated with patient survival using Cox proportional hazards models, with piRNA expression in tumors (cpm) modeled as a binary variable based on expression above or below the median. Survival analyses were independently conducted on HPV16(+) HNSCC patients, HPV16(+) Smoking patients, and HPV16(+) Nonsmoking cohorts. We first performed univariate Kaplan-Meier analysis and univariate Cox regression analysis to identify candidates significantly associated with patient outcome (*p* < 0.05), and then performed multivariate Cox analysis to evaluate whether correlations were independent of clinical variables such as age (grouped into 10-year intervals), gender, and tumor grade and stage.

### Cell culture

HPV(+) squamous cell carcinoma cell lines UM-SCC-47, derived from the primary tumor of the lateral tongue of a male patient, and 93-VU-147T, derived from the upper aerodigestive tract, were generous gifts from Dr. Joseph Califano at the University of California, San Diego School of Medicine. HaCaT, a spontaneously transformed immortal keratinocyte cell line derived from human skin, was a generous gift from Dr. Victor Nizet at the Center for Immunity, Infection, and Inflammation of the UC San Diego School of Medicine. These cells were cultured in DMEM supplemented with 10% fetal bovine serum, 2% penicillin/streptomycin, and 2% L-glutamate (GIBCO) and maintained at 37°C in a humidified 5% CO_2_/95% air atmosphere.

Normal, early passage, oral epithelial cell line OKF6, derived from the floor of the mouth of genetically and clinically normal tissue, was a generous gift from the Rheinwald Lab at Harvard Medical School. The cells were cultured in keratinocyte serum-free media (Life Technologies) supplemented with EGF, bovine pituitary extract, 2% L-glutamine, 2% penicillin/streptomycin, and CaCl_2_ and maintained at 37°C in a humidified 5% CO_2_/95% air atmosphere.

### Quantification of piRNA expression by qRT-PCR

Total RNA was isolated (Fisher Scientific) from cultured cells. cDNA was synthesized using the QuantiMiR^™^ RT kit (System Biosciences) as per the manufacturer's instructions. Real-time PCR reaction mixes were created using FastStart Universal SYBR Green Master Mix (Roche Diagnostics), and run on a StepOnePlus^™^ Real-Time PCR System (Applied Biosystems) using the following program: 50°C for 2 min, 95°C for 10 min, 95°C for 30 s, and 60°C for 1 min, for 40 cycles. Experiments were analyzed using the ddCt method. U6 primers and a Universal Reverse Primer were used from the QuantiMiR^™^ RT kit, and custom primers (Integrated DNA Technologies) were ordered using the following sequences: *NONHSAT077364*: 5′ – ACCTGATGTTACATTGTAGTGTGCTGATG – 3′, *NONHSAT102574*: 5′ – CATGATACTGTAAACGCTTT CTGATG – 3′, *NONHSAT128479*: 5′ – GGCTCTGTTGC GCAATGGATAGCGCAT – 3′.

## SUPPLEMENTARY MATERIALS TABLES









## Abbreviations

HNSCC: head and neck squamous cell carcinoma
HPV: human papillomavirus
ncRNA: non-coding RNA
piRNA: PIWI-interacting RNA
TCGA: The Cancer Genome Atlas
GSEA: Gene set enrichment analysis

## References

[1] Marur S, D'Souza G, Westra WH, Forastiere AA (2010). HPV-associated head and neck cancer: a virus-related cancer epidemic. Lancet Oncol.

[2] Agalliu I, Gapstur S, Chen Z, Wang T, Anderson RL, Teras L, Kreimer AR, Hayes RB, Freedman ND, Burk RD (2016). Associations of Oral α-, β-, and γ-Human Papillomavirus Types With Risk of Incident Head and Neck Cancer. JAMA Oncol.

[3] Gillison ML, Koch WM, Capone RB, Spafford M, Westra WH, Wu L, Zahurak ML, Daniel RW, Viglione M, Symer DE, Shah KV, Sidransky D (2000). Evidence for a causal association between human papillomavirus and a subset of head and neck cancers. J Natl Cancer Inst.

[4] Narisawa-Saito M, Kiyono T (2007). Basic mechanisms of high-risk human papillomavirus-induced carcinogenesis: roles of E6 and E7 proteins. Cancer Sci.

[5] Calore F, Lovat F, Garofalo M (2013). Non-coding RNAs and cancer. Int J Mol Sci.

[6] Huang T, Alvarez A, Hu B, Cheng SY (2013). Noncoding RNAs in cancer and cancer stem cells. Chin J Cancer.

[7] Huarte M (2015). The emerging role of lncRNAs in cancer. Nat Med.

[8] Sasaki T, Shiohama A, Minoshima S, Shimizu N (2003). Identification of eight members of the Argonaute family in the human genome. Genomics.

[9] Meister G (2013). Argonaute proteins: functional insights and emerging roles. Nat Rev Genet.

[10] Zou AE, Zheng H, Saad MA, Rahimy M, Ku J, Kuo SZ, Honda TK, Wang-Rodriguez J, Xuan Y, Korrapati A, Yu V, Singh P, Grandis JR (2016). The non-coding landscape of head and neck squamous cell carcinoma. Oncotarget.

[11] Krishnan AR, Korrapati A, Zou AE, Qu Y, Wang XQ, Califano JA, Wang-Rodriguez J, Lippman SM, Hovell MF, Ongkeko WM (2017). Smoking status regulates a novel panel of PIWI-interacting RNAs in head and neck squamous cell carcinoma. Oral Oncol.

[12] Ng KW, Anderson C, Marshall EA, Minatel BC, Enfield KS, Saprunoff HL, Lam WL, Martinez VD (2016). Piwi-interacting RNAs in cancer: emerging functions and clinical utility. Mol Cancer.

[13] Bourc’his D, Bestor TH (2004). Meiotic catastrophe and retrotransposon reactivation in male germ cells lacking Dnmt3L. Nature.

[14] Ross RJ, Weiner MM, Lin H (2014). PIWI proteins and PIWI-interacting RNAs in the soma. Nature.

[15] Wang Z, Liu N, Shi S, Liu S, Lin H (2016). The Role of PIWIL4, an Argonaute Family Protein, in Breast Cancer. J Biol Chem.

[16] Aravin AA, Bourc’his D (2008). Small RNA guides for de novo DNA methylation in mammalian germ cells. Genes Dev.

[17] Aravin AA, Sachidanandam R, Bourc’his D, Schaefer C, Pezic D, Toth KF, Bestor T, Hannon GJ (2008). A piRNA pathway primed by individual transposons is linked to de novo DNA methylation in mice. Mol Cell.

[18] Watanabe T, Lin H (2014). Posttranscriptional regulation of gene expression by Piwi proteins and piRNAs. Mol Cell.

[19] Brandt J, Veith AM, Volff JN (2005). A family of neofunctionalized Ty3/gypsy retrotransposon genes in mammalian genomes. Cytogenet Genome Res.

[20] Youngson NA, Kocialkowski S, Peel N, Ferguson-Smith AC (2005). A small family of sushi-class retrotransposon-derived genes in mammals and their relation to genomic imprinting. J Mol Evol.

[21] Nagasaki K, Manabe T, Hanzawa H, Maass N, Tsukada T, Yamaguchi K (1999). Identification of a novel gene, LDOC1, down-regulated in cancer cell lines. Cancer Lett.

[22] Riordan JD, Keng VW, Tschida BR, Scheetz TE, Bell JB, Podetz-Pedersen KM, Moser CD, Copeland NG, Jenkins NA, Roberts LR, Largaespada DA, Dupuy AJ (2013). Identification of rtl1, a retrotransposon-derived imprinted gene, as a novel driver of hepatocarcinogenesis. PLoS Genet.

[23] Bian Y, Hall B, Sun ZJ, Molinolo A, Chen W, Gutkind JS, Waes CV, Kulkarni AB (2012). Loss of TGF-beta signaling and PTEN promotes head and neck squamous cell carcinoma through cellular senescence evasion and cancer-related inflammation. Oncogene.

[24] Kulke MH, Odze RD, Thakore KS, Thomas G, Wang H, Loda M, Eng C (2001). Allelic loss of 10q23, the PTEN tumour suppressor gene locus, in Barrett's oesophagus-associated adenocarcinoma. Br J Cancer.

[25] Squarize CH, Castilho RM, Abrahao AC, Molinolo A, Lingen MW, Gutkind JS (2013). PTEN deficiency contributes to the development and progression of head and neck cancer. Neoplasia.

[26] Surviladze Z, Sterk RT, DeHaro SA, Ozbun MA (2013). Cellular entry of human papillomavirus type 16 involves activation of the phosphatidylinositol 3-kinase/Akt/mTOR pathway and inhibition of autophagy. J Virol.

[27] Lobry C, Oh P, Aifantis I (2011). Oncogenic and tumor suppressor functions of Notch in cancer: it's NOTCH what you think. J Exp Med.

[28] Stransky N, Egloff AM, Tward AD, Kostic AD, Cibulskis K, Sivachenko A, Kryukov GV, Lawrence MS, Sougnez C, McKenna A, Shefler E, Ramos AH, Stojanov P (2011). The mutational landscape of head and neck squamous cell carcinoma. Science.

[29] Beck TN, Golemis EA (2016). Genomic insights into head and neck cancer. Cancers Head Neck.

[30] Populo H, Lopes JM, Soares P (2012). The mTOR signalling pathway in human cancer. Int J Mol Sci.

[31] Lin M, Smith LT, Smiraglia DJ, Kazhiyur-Mannar R, Lang JC, Schuller DE, Kornacker K, Wenger R, Plass C (2006). DNA copy number gains in head and neck squamous cell carcinoma. Oncogene.

[32] Subramanian A, Tamayo P, Mootha VK, Mukherjee S, Ebert BL, Gillette MA, Paulovich A, Pomeroy SL, Golub TR, Lander ES, Mesirov JP (2005). Gene set enrichment analysis: a knowledge-based approach for interpreting genome-wide expression profiles. Proc Natl Acad Sci U S A.

[33] Enright AJ, John B, Gaul U, Tuschl T, Sander C, Marks DS (2003). MicroRNA targets in Drosophila. Genome Biol.

