# Supplementary material for: Computational methods reveal novel functionalities of PIWI-interacting RNAs in human papillomavirus-induced head and neck squamous cell carcinoma
Source: Oncotarget. 2017 Dec 19;9(4):4614–24. doi: 10.18632/oncotarget.23464 (PMC5797000; doi:10.18632/oncotarget.23464)
Supplement: Supplementary file 1 [file oncotarget-09-4614-s001.pdf]

Computational methods reveal novel functionalities of PIWI-interacting RNAs in human papillomavirus-induced head and neck squamous cell carcinoma

SUPPLEMENTARY MATERIALS

Supplementary Table 1: TCGA dataset IDs. See Supplementary_Table_1

Supplementary Table 2: Dysregulated piRNAs between HPV(+) and HPV(–) cohorts. See Supplementary_Table_2

Supplementary Table 3A: Frequent somatic mutations in HNSCC

CASP8
CDKN2A
DDX3X
DICER1
EGFR
EZH2
FAT1
FBXW7
HRAS
IRF6
KMT2D
MED1
NOTCH1
NOTCH2
NOTCH
PCLO
PIK3CA
PRDM9
PTEN
RB1
RIMS2
RIPK4
SYNE1
SYNE2
TP53
TP63

Supplementary Table 3B: Frequent Copy Number Variations in HNSCC. See Supplementary_Table_3B

Supplementary Table 4: Multivariate cox regression analysis results for NONHSAT077364

	HR	Lower 95%	Upper 95%	*p*-value
NONHSAT077364 (Low vs. High)	2.15E–01	4.87E–02	9.45E–01	0.0419
Race (White vs. Black or African American)	5.10E–02	2.42E–03	1.0745	0.0556
Age	9.58E–01	8.76E–01	1.0476	0.347
Gender (Male vs. Female)	4.49E+00	1.90E–02	1.06E+03	0.5901
Histologic Grade (relative to G1)				
G2	1.35E+08	0.00E+00	Inf	0.9982
G3	3.59E+07	0.00E+00	Inf	0.9983
G4	4.32E–01	0.00E+00	Inf	1
Clinical N Stage (relative to N0)				
N1	6.74E–02	3.08E–03	1.47	0.0866
N2	8.84E–01	4.05E–02	19.29	0.9374
N3	1.13E+00	8.39E–02	6.54	0.7872
